# *In vivo* Diffusion Tensor Magnetic Resonance Tractography of the Sheep Brain: An Atlas of the Ovine White Matter Fiber Bundles

**DOI:** 10.3389/fvets.2019.00345

**Published:** 2019-10-16

**Authors:** Valentina Pieri, Marco Trovatelli, Marcello Cadioli, Davide Danilo Zani, Stefano Brizzola, Giuliano Ravasio, Fabio Acocella, Mauro Di Giancamillo, Luca Malfassi, Mario Dolera, Marco Riva, Lorenzo Bello, Andrea Falini, Antonella Castellano

**Affiliations:** ^1^Neuroradiology Unit and CERMAC, Vita-Salute San Raffaele University, IRCCS San Raffaele Scientific Institute, Milan, Italy; ^2^Department of Health, Animal Science and Food Safety, Faculty of Veterinary Medicine, University of Milan, Milan, Italy; ^3^Philips Healthcare, Milan, Italy; ^4^Department of Veterinary Medicine, Università degli Studi di Milano, Milan, Italy; ^5^Fondazione La Cittadina Studi e Ricerche Veterinarie, Romanengo, Italy; ^6^Department of Medical Biotechnology and Translational Medicine, Università degli Studi di Milano, Milan, Italy; ^7^Neurosurgical Oncology Unit, Humanitas Clinical and Research Center – IRCCS, Rozzano, Italy; ^8^Department of Oncology and Hemato-Oncology, Università degli Studi di Milano, Milan, Italy

**Keywords:** diffusion tensor imaging, DTI tractography, sheep, brain, atlas

## Abstract

Diffusion Tensor Magnetic Resonance Imaging (DTI) allows to decode the mobility of water molecules in cerebral tissue, which is highly directional along myelinated fibers. By integrating the direction of highest water diffusion through the tissue, DTI Tractography enables a non-invasive dissection of brain fiber bundles. As such, this technique is a unique probe for *in vivo* characterization of white matter architecture. Unraveling the principal brain texture features of preclinical models that are advantageously exploited in experimental neuroscience is crucial to correctly evaluate investigational findings and to correlate them with real clinical scenarios. Although structurally similar to the human brain, the gyrencephalic ovine model has not yet been characterized by a systematic DTI study. Here we present the first *in vivo* sheep (*ovis aries*) tractography atlas, where the course of the main white matter fiber bundles of the ovine brain has been reconstructed. In the context of the EU's Horizon EDEN2020 project, *in vivo* brain MRI protocol for ovine animal models was optimized on a 1.5T scanner. High resolution conventional MRI scans and DTI sequences (b-value = 1,000 s/mm^2^, 15 directions) were acquired on ten anesthetized sheep *o. aries*, in order to define the diffusion features of normal adult ovine brain tissue. Topography of the ovine cortex was studied and DTI maps were derived, to perform DTI tractography reconstruction of the corticospinal tract, corpus callosum, fornix, visual pathway, and occipitofrontal fascicle, bilaterally for all the animals. Binary masks of the tracts were then coregistered and reported in the space of a standard stereotaxic ovine reference system, to demonstrate the consistency of the fiber bundles and the minimal inter-subject variability in a unique tractography atlas. Our results determine the feasibility of a protocol to perform *in vivo* DTI tractography of the sheep, providing a reliable reconstruction and 3D rendering of major ovine fiber tracts underlying different neurological functions. Estimation of fiber directions and interactions would lead to a more comprehensive understanding of the sheep's brain anatomy, potentially exploitable in preclinical experiments, thus representing a precious tool for veterinaries and researchers.

## Introduction

The organization of white matter (WM) microstructure and brain connections can be depicted *in vivo* by Diffusion Tensor Imaging (DTI), an advanced Magnetic Resonance (MR) technique which provides a unique probe to non-invasively quantify the directional, *anisotropic* mobility of water molecules within tissues (1). In cerebral WM, water mobility is hindered along the main direction of the fibers by the multiple layers of myelin around the axons; therefore, the principal axis of the diffusion tensor aligns with the predominant fiber orientation within the tissue (2, 3). One of the most important applications of DTI technique is MR tractography, or fiber-tracking, a method that can be used for the virtual dissection of the relevant myelin-sheathed fiber bundles within the brain. MR tractography is the process of integrating fiber orientations within tissue into a trajectory connecting remote brain areas, starting from anatomical seed regions and following the directions of highest water diffusion until stopping criteria are met (4).

DTI and tractography have broadened the understanding of the WM pathways concealed within the brain tissue, complementing anatomical information from conventional MRI. Furthermore, the increasing exploitation of these technique in clinical studies has revealed their great potential for understanding healthy and pathological brain anatomy in humans, paving the way toward their application also in preclinical animal models. Therefore, translational researchers are increasingly embracing these techniques for a comprehensive description of white matter fiber distribution in healthy animals as well as to study developmental pathologies, exposure to teratogens, or the effects of malnutrition and hypoxia in the prenatal environment (5).

Remarkably, the evaluation of brain structural and ultrastructural features with DTI in preclinical models offers the possibility of validating the imaging-derived information by comparing them to histological sections with precise spatial correlation, once the animals have been sacrificed. Different animal models have been investigated with DTI, such as ferrets (6, 7), rats (8, 9), tree shrew (10), and mice (11, 12). Nonetheless, it is worth of note that most of DTI studies on larger animals have been performed on formalin-fixed brain sections and not *in vivo*, thus potentially invalidating the diffusion properties of brain tissue. This has been the case of studies on non-human primates (13–15), dolphins (16), dogs (17, 18), and cats (19).

On the other hand, the application of DTI and tractography has been extremely deficient in other species considered as relevant models in translational research, including sheep. Sheep offer a great degree of research translatability into basic brain functions as they have a long lifespan, have rudimentary and well-established housing demands and are safer than primates to be managed in experimental settings, especially because they do not have hands to interfere with equipment (20). Most importantly, the gyrencephalic ovine brain is anatomically and functionally more similar to human brain if compared to the lissencephalic brain of rodents and rabbits, due to its relatively large size and the presence of sulci (21). Further similar features to humans are evident in electroencephalographic records, neuroradiological features, and neurovascular structures (22), thus making the ovine model of particular relevance in the field of experimental neuroscience. For example, it has been studied in the context of epilepsy (23), neuropsychiatry (24), traumatic brain injury (25), and neurodegenerative diseases (26, 27). The similarities between caudate, putamen and substantia nigra in the sheep and human brains, in fact, provide a valuable and valid tool for modeling basal ganglia diseases (28). Intriguingly, studies recently demonstrated neurofibrillary accumulation in normal aged sheep, extremely similar to the tau deposits associated to Alzheimer's disease (AD) in humans, so that researchers are now testing the usefulness of the ovine for future genetic manipulation to generate AD animal model (27).

Furthermore, due to these similarities with human brain, the ovine model can be particularly valuable in the context of neurosurgical research to test new devices and peri-operative technologies. In this scenario, the possibility to explore *in vivo* the imaging features of the ovine brain becomes relevant for pre-surgical planning and intraoperative neuronavigation. Despite the growing interest in using sheep as a model of large mammals with complex central nervous system, comprehensive ovine MRI studies are rather limited, presumably because they are hardly feasible and require a well-organized and specialized multidisciplinary team (29). The main efforts have been focused on defining MRI templates and atlases of the ovine brain based on conventional T1- and T2-weighted MR images, and to build up a standard stereotaxic ovine reference system (30–32). On the other hand, advanced MR imaging studies including DTI on sheep are even rarer and on a small number of animals. To our knowledge, the only work on the applicability of DTI on living healthy sheep has been conducted by Lee et al., on a limited sample of 6 sheep (33). They exploited functional MRI (fMRI) to visualize the sensorimotor and visual cortex activated by external sensory stimuli, then performed DTI tractography to reconstruct corticospinal tract and optic radiations starting from the activated cortical areas by performing single-subject analysis (33). Despite representing a step forward for demonstrating the feasibility of advanced MRI of the ovine models, this study was focused only on two fiber bundles. Thus, a detailed analysis of the ovine white matter organization using DTI is still lacking, and an ovine population-averaged MR tractography atlas within a standard stereotaxic reference system (31) has not been reported yet. Hence, the aims of the present work are to demonstrate the reproducibility of DTI tractography in living sheep by performing a comprehensive reconstruction of five main white matter fiber bundles of the ovine brain, and to create the first *in vivo*, population-averaged sheep MR tractography atlas integrated within a standard stereotaxic ovine reference system.

## Materials and Methods

### Study Population and Ethics

A total of 10 adult female sheep *ovis aries* (Bergamasca, weight = 72.2 ± 5.4 kg) were used in this study, carried on in the context of the European Union's EU Horizon EDEN2020 project (https://www.eden2020.eu/) that has the final aim of testing an integrated technology platform for minimally invasive brain surgery on ovine models. Sheep have been selected due to their anatomy, physiology, and neurological development. The choice of female gender was due to the size, weight and to the social behavior characterized by a low agonist component, favoring housing and handling. For all subjects, a detailed clinical and physiological score was daily reported in an *ad-hoc* ethogram (Supplementary Table 1). The sheep were fasted but with free access to water 24 h prior to each imaging session. All animals were treated in accordance with the European Communities Council directive (2010/63/EU), to the laws and regulations on animal welfare enclosed in D.L.G.S. 26/2014. Ethical approval for this study was obtained by the Italian Health Department with authorization n 635/2017.

### Anesthesia and *in vivo* MR Imaging

Animals were anesthetized via the intravenous administration of Diazepam 0.25 mg/Kg + Ketamine 5 mg/Kg, intubated and then maintained under general anesthesia with isoflurane 2% and oxygen 2 L/min. They were transported to the imaging facility and placed in prone position, with the head located in a MRI-compatible headframe (Renishaw®) specifically made for the EDEN2020 project. MR imaging was performed on a 1.5T clinical scanner (Achieva, Philips Healthcare) in a veterinary imaging facility [Fondazione La Cittadina Studi e Ricerche Veterinarie, Romanengo (CR), Italy]. Small and medium flex coils fixed over both hemispheres were used. Diffusion Tensor Imaging (DTI) data were obtained from all the animals by using a single-shot echo planar sequence with parallel imaging (SENSE factor *R* = 2). Diffusion gradients were applied along 15 non-collinear directions, using a b-value of 1,000 s/mm^2^. The detailed imaging parameters for DTI were: TR/TE 6,700 ms/84 ms; acquisition isotropic voxel size 2 × 2 × 2 mm; acquisition matrix 96 × 96; FOV 192 × 192 mm; slice thickness 2 mm; 45 contiguous slices without gap. Two signal averages (NSA = 2) were obtained, for a total scan time of 5 min 34 s. A T1-weighted volumetric scan was also acquired from each animal by using a three-dimensional fast-field-echo (3D-T1 FFE) sequence with the following parameters: TR/TE 25/5 ms; flip angle 40°; voxel size 0.667 × 0.667 × 1.4 mm; SENSE factor *R* = 2; 150 slices; acquisition time 8 min 40 s. All the MRI sequences were oriented perpendicular to the longitudinal axis of the scanner without rotation in any plane, in order to minimize the requested steps for coregistration. Other structural/anatomical MR imaging sequences were acquired for purpose of neuronavigation in the context of the EDEN2020 project, but they were not used for the herein presented study. The detailed parameters of these sequences are listed in Supplementary Table 2.

### DTI Data Analysis and Tractography Reconstructions

**Figure 1:**
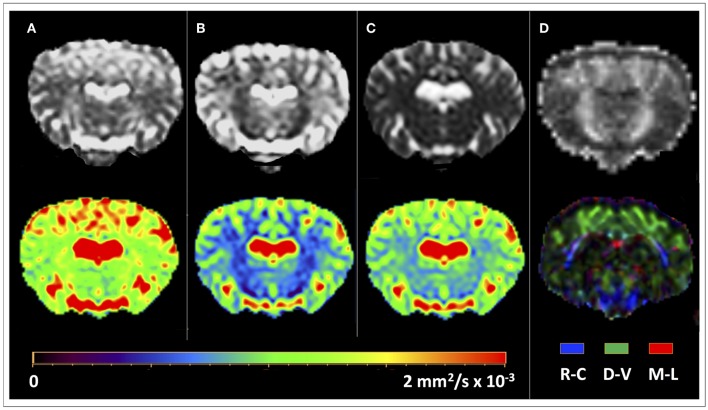
DTI-derived maps for a representative ovine brain. Computed maps of diffusion (mm^2^/s × 10^−3^) are shown in gray scale (upper row) and color LUT (Look Up Tables) display (lower row). **(A)** Axial Diffusivity (AD) map. **(B)** Radial Diffusivity (RD) map; notably, low values in correspondence of the ovine internal capsule indicate the dominance of the principal direction in that region. **(C)** Mean Diffusivity (MD) map. **(D)** Fractional Anisotropy (FA) map. Color-coded FA maps (lower row) display fibers with rostral-caudal (R-C) direction in blue, fibers with dorsal-ventral (D-V) direction in green, and fibers with medial-lateral (M-L) in red.

**Figure 2:**
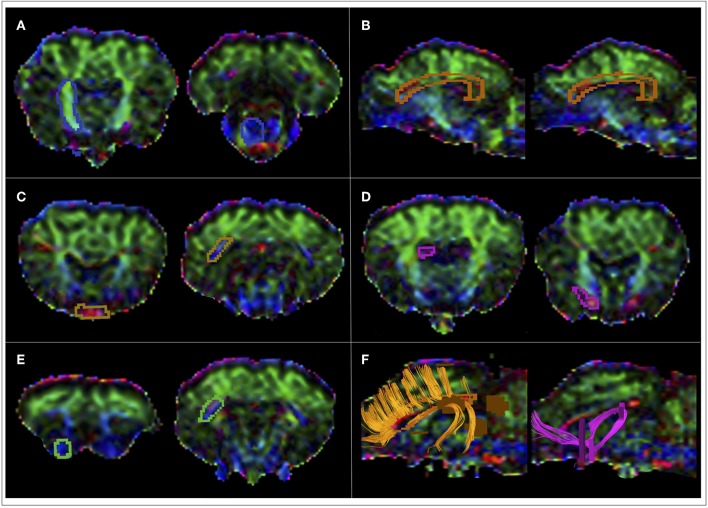
Location of the ROIs for tractography reconstructions. Seed regions-of-interest (ROIs) were contoured in precise anatomical positions selected to encompass the tract cross-section, according to the location of different fiber tracts. **(A)** ROIs for CST tractography reconstruction were placed on the transverse plane. The first ROI was placed in the genu of the internal capsule, that it is known to comprehend the ovine pyramidal tract and is found between the caudate nucleus and the putamen. The other ROI included the ventral mesencephalic plane, where the pons is located. **(B)** ROIs for CC tractography reconstruction were placed on the sagittal plane. ROIs were carefully positioned on two slices to contain a section of the fiber tract of interest in 3D space. In particular, the corpus callosum at the level of septum pellucidum was contoured by a ROI extending from the infrasplenial sulcus to the genual sulcus. **(C)** ROIs for VP tractography reconstruction were placed on the transverse plane. The ROI including the chiasm was set between the rostral encephalic longitudinal fissure and the posterior tuber cinereum. The optic radiations are known to run in the retrolentiform portion of the internal capsule and to diverge caudally before reaching the occipital cortex. Thus, a ROI has been placed just above the caudal portion of the lateral ventricle. **(D)** ROIs for FX tractography reconstruction were placed on the transverse plane. The first ROI encompassed the white matter medial to the genu of the internal capsule, rostral to the anterior horns of the lateral ventricles. The other ROI has been contoured posteriorly to the optic chiasm, external to the rostral arm of the internal capsule. **(E)** ROIs for OF tractography reconstruction were placed on the transverse plane. The rostral ROI was placed in the orbital gyrus, just posterior to the olfactory bulbs. The caudal ROI has been placed just above the caudal portion of the lateral ventricle, exactly as for the optic radiations that are known to be closely surrounded by the OF fibers. **(F)** Example of raw CC and FX tracts resulting from the first tracking procedures, that were successively refined by inserting exclusion ROIs that allowed the removal of spurious fibers.

DTI data were analyzed with the Philips IntelliSpace Portal software platform, version 8.0 (Philips Healthcare, Best, The Netherlands). DTI datasets were firstly corrected for motion artifacts by applying affine alignment of each diffusion-weighted image to the b = 0 image. Then, the DTI of each sheep was coregistered to the reference image 3DT1 of the same sheep by means of a 3-D affine transformation based on local correlation similarity (34), using the Local Correlation tool of the Philips IntelliSpace Portal software. Diffusion tensor element were calculated and diagonalized at each voxel using the MR Diffusion tool of the Intellispace Portal software platform, obtaining the three eigenvectors (ε_1_, ε_2_, ε_3_), and diffusivities (λ_1_, λ_2_, λ_3_) along these vectors. From these, Mean Diffusivity (MD), Axial Diffusivity (AD), Radial Diffusivity (RD), and Fractional Anisotropy (FA) maps were computed for each sheep (Figure 1). MD is a measure of the orientation-averaged apparent diffusivity within a voxel, while AD and RD, respectively measure water diffusion parallel and perpendicular to the main axis of the tensor. The Fractional Anisotropy (FA) map describes the degree of diffusion anisotropy within a voxel, being a scalar value between 0 and 1. From the combination of the FA and principal eigenvector ε_1_, color maps were generated with conventional color-coding (35). Color-coded FA maps emphasizes the directionality of diffusion by displaying fibers with rostral-caudal (R-C) direction in blue, fibers with dorsal-ventral (D-V) direction in green, and fibers with medial-lateral (M-L) in red (Figure 1D). Whole brain deterministic tractography was performed using the MR Fiber Trak tool of the IntelliSpace Portal software through the fiber assignment by continuous tracking algorithm, with an FA threshold of 0.15 and an angle threshold of 27°. Inclusive and exclusive seed regions-of-interest (ROIs) were manually delineated on the color-coded FA maps for a virtual dissection of the main ovine WM tracts. The identification of the main anatomical structures in the sheep was facilitated by the fact that color-coded FA maps were coregistered to high resolution T1-weighted volumetric reference images. For each tract, seed ROIs were placed in different anatomical positions according to the different fiber tracts *in consensus* by an expert neuroradiologist (A.C. with 15 years of experience in DTI tractography reconstructions), a PhD student in neuroimaging (V.P., with 2 years of experience in DTI tractography analysis) and a PhD student in veterinary sciences with a specific training on anatomical dissection of ovine brain (M.T., with 4 years of experience). WM tracts were identified on the basis of the ovine neuroanatomical literature (36–40), human DTI atlas (41, 42), and gross dissections of ovine brain (43). ROIs were selected to encompass the tract cross-section and were labeled as *inclusive* for start tracking, both in forward ad in backward directions (Figures 2A–E). Raw tracts resulting from the first tracking procedure were then refined on the basis of anatomical knowledge, by removing eventual contaminating fibers with *exclusion* ROIs (Figure 2F). Five eloquent WM tracts were reconstructed bilaterally in each sheep: corticospinal tract (CST), corpus callosum (CC), visual pathway (VP), fornix (FX), and occipitofrontal fasciculus (OF). These tracts were chosen as they represent the main fiber bundles of the ovine brain, that can be reconstructed for neurosurgical planning purposes in typical *in vivo* DTI studies. Fiber tracts were finally displayed as volumes in different colors, and were overlaid as binary masks onto the T1-weighted volumetric images of each sheep, on which the DTI sequence was previously coregistered. These binary images were saved in the Surgical Navigation-compatible DICOM (Digital Imaging and Computing in Medicine) format of the Intellispace software.

### Ovine Brain Tractography Atlas Creation

For each sheep, the DICOM images of the T1-weighted volumetric series and of the coregistered tracts' binary masks were converted to the NIfTI (Neuroimaging Informatics Technology Initiative) format using the “dcm2nii” tool (https://people.cas.sc.edu/rorden/mricron/dcm2nii.html). Ovine brain masks were obtained by using the FMRIB Software Library (FSL, University of Oxford, https://fsl.fmrib.ox.ac.uk/fsl/) brain extraction tool (BET), setting fractional intensity threshold function at 0.4, and manually refined with ITK-SNAP software (version 3.6.0). They were eventually multiplied to the original T1-weighted volumetric images using the “fslmaths” tool of FSL, in order to extract the whole brain of each sheep. Then, the original T1-weighted volumetric series of each sheep was coregistered to the publicly available T1-weighted stereotaxic ovine brain template developed by Nitzsche et al. (31) by using an affine transformation with the FLIRT tool of FMRIB Software Library (FSL, https://fsl.fmrib.ox.ac.uk/fsl/). The derived transformation matrix was consequently applied to all the reconstructed tracts. All the tracts in the stereotaxic atlas space were finally converted into binary mask images using ITK-SNAP (V3.6.0) software (http://www.itksnap.org/pmwiki/pmwiki.php). In each mask image, the voxels containing at least one streamline of the tract are associated with value 1, the others with value 0. The binary mask images of each tract in the stereotaxic space were eventually summed by means of the “fslmaths” function of FSL into a single mask, representing voxel-by-voxel probability of the presence of the tract in the 10 animals, thus ranged between 0 and 10.

### Descriptive Statistics of Quantitative Diffusion Metrics in Fiber Tracts

With the aim of quantifying the principal diffusion metrics in fiber bundles, average values of tract-specific Fractional Anisotropy (FA: scalar value 0-1) and Mean Diffusivity (MD: mm^2^/s × 10^−3^) were calculated for each sheep by means of the Tract Statistical extraction tool of IntelliSpace Portal software platform, version 8.0. Afterwards, data of all the 10 CST, 10 CC, 10 VP, 10 FX, and 10 OF were analyzed, in order to obtain population-averaged diffusion values of all the five WM tracts. D'Agostino & Pearson normality test was applied to assess data distribution, then minimum, maximum, and mean ± SD values of FA and MD were computed and displayed in box-plots to describe the distribution of diffusion metrics within our sample.

## Results

### DTI Tractography Analysis in Individual Sheep

**Figure 3:**
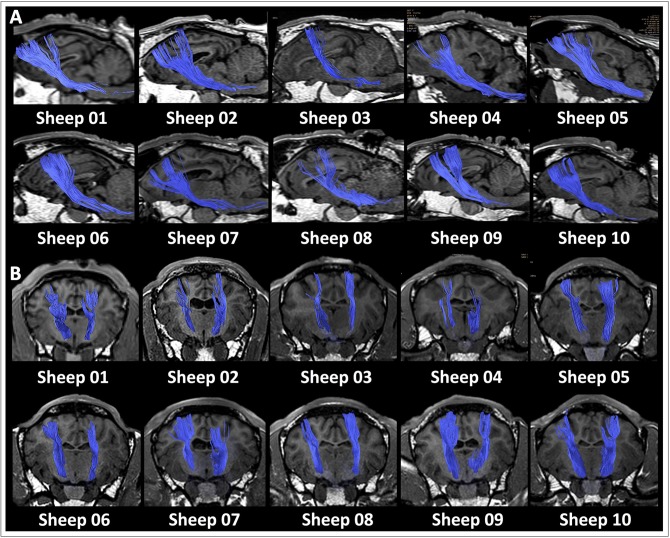
Reproducibility of CST tractography reconstruction in all the 10 sheep. The anatomical course of the corticospinal tract (CST, blue) is shown as three-dimensional rendering in a sagittal **(A)** and frontal **(B)** view for each of the 10 sheep. At a qualitative, visual assessment the course of tracts appears very similar across the 10 animals, thus highlighting the reproducibility of the tractography pipeline.

DTI data acquisition was performed in all the ovine models, and DTI datasets were successfully post-processed in all the cases, allowing the *in vivo* dissection of the main projection, associative and commissural fibers in the ovine brain. Reconstruction of the corticospinal tract (CST), corpus callosum (CC), visual pathway (VP), fornix (FX) and occipitofrontal fasciculus (OF) was feasible in all the 10 sheep. A detailed description of seed ROIs' position for each tract has been reported in Figure 2. The course of tracts was reproducible and consistent across all the animals. Figure 3 shows an example of the whole anatomical course of the CST both in the sagittal and frontal view for each of the 10 sheep, revealing a minimal inter-sheep variability at a qualitative, visual assessment.

#### Corticospinal Tract (CST)

**Figure 4:**
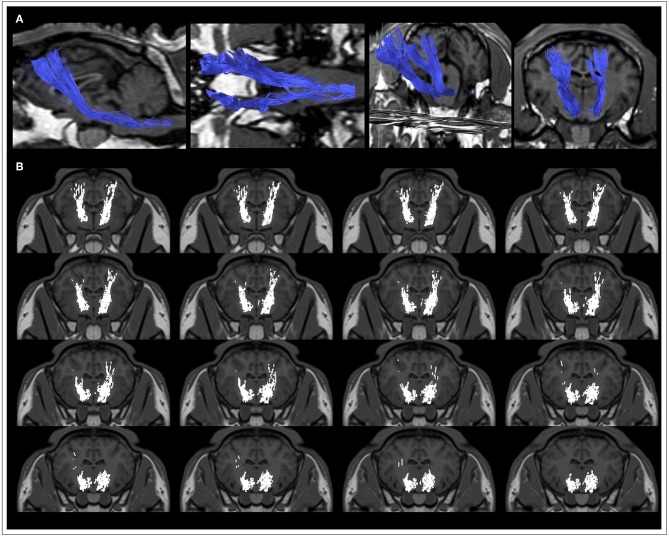
Details of the course of the CST in a representative sheep. **(A)** CSTs are displayed as volumes on the corresponding T1-weighted anatomical image. **(B)** CSTs are overlaid as binary masks onto the T1-weighted volumetric images.

The ovine primary motor cortex lays in the precruciate gyrus, immediately anterior to the cruciate fissure, and the CST is the main efferent bundle that connects it to the spinal cord (Figures 3, 4). Along its course, the CST reaches the corona radiata and its fibers intersect the radiation of the corpus callosum at the level of centrum semiovale. Then, it passes through the posterior limb of the internal capsule and continues to the medial portion of cerebral peduncle, reaching the lateral funiculus. After the pontine nuclei, the white matter bundles converge in the pyramidal tract on the ventral bulbi surface.

#### Corpus Callosum (CC)

**Figure 5:**
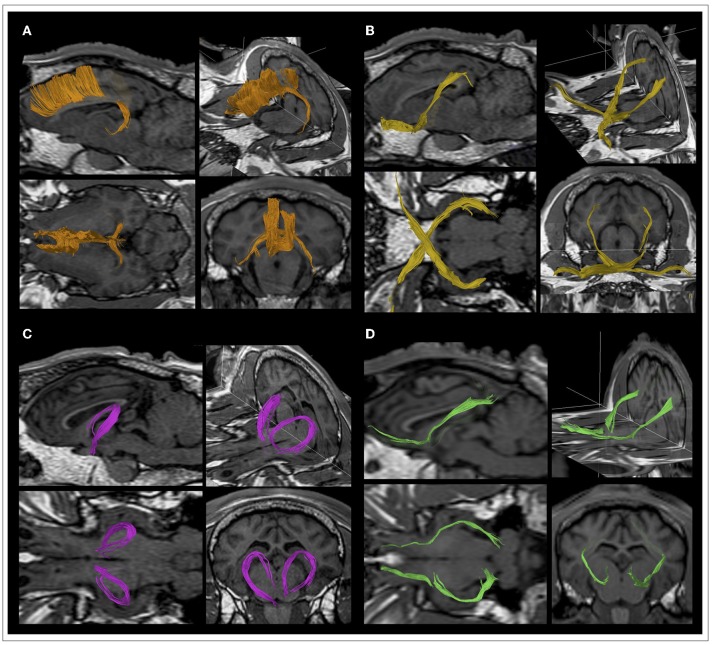
Details of the course of the main ovine white matter fiber bundles in a representative sheep. Tracts are displayed as volumes onto high-resolution T1 anatomical images and showed in sagittal, lateral, coronal and transverse views. **(A)** Corpus callosum (CC, orange). **(B)** Visual pathway (VP, yellow). **(C)** Fornix (FX, pink). **(D)** Occipitofrontal fasciculus (OF, green).

The ovine CC is a large band of fibers constituting the main commissural structure of the brain and it links homologous frontal motor areas from the two different hemispheres. Its medial part, the body, is located along the brain midline and forms the roof of large part of the lateral ventricles, then dispersing its fibers at the level of centrum semiovale, crossing the corona radiata. Anteriorly, the genu of CC is identifiable as sharply bent white matter bundles, that narrow into the rostrum as the genu turns under. It connects the frontal cortex bilaterally, generating an arch path known as forceps minor. Posteriorly, the CC ends in the splenium: fiber bundles reach the parieto-occipital lobes bilaterally, generating an arch path known as forceps major that links parietal sensitive homologs areas. The callosal sulcus separates the CC from the adjacent midline cortex, the cingulate gyrus (Figure 5A).

#### Visual Pathway (VP)

The VP in ovine crosses the brain rostro-caudally. Initially, the optic nerve connects the retina to the optic chiasm, which is surrounded by the Circle of Willis, allowing communications between the carotid and vertebral or basilar supplies to the brain. From the chiasm, optic tracts depart passing lateral to the cerebral peduncles and ending in the thalamic lateral geniculate nuclei (LGN). These nuclei constitute the thalamic receiving area for vision and are sidelong the medial geniculates, the thalamic relay nuclei for auditory fibers. From LGN, fibers are conveyed via the geniculocalcarine tract or optic radiation through the sublenticular portion of the internal capsule, and then to the primary visual cortex in the occipital lobe (Figure 5B).

#### Fornix (FX)

The FX is composed by an arch of fibers arising from the fimbria below the posterior portion of the corpus callosum and bending downward to dive below the surface, in route to the mammillary bodies. The FX constitutes part of the dorsal and rostral limit of the thalamus and is divided by a commissure which connects the two hippocampi, named commissure of fornix. Caudally, it is composed by two stripes of white matter that are known as fornix legs and are the extensions of the hippocampus fimbriae. Fornix legs continue rostrally forming the body of the fornix, which then creates two different ropes named fornix columns. The fornix columns proceed ventrally, ending the pathway into the corpi mamillari. The rostral arch of the fornix is adjacent to the anterior commissure, which connects the olfactory bulb, pyriform area, and amygdala of the two hemispheres (Figure 5C).

#### Occipitofrontal Fasciculus (OF)

The OF connects the frontal with the occipital lobe, starting rostral to the olfactory bulbs. It is found along the lateral border of the caudate nucleus and on the lateral aspect of the CST. After passing near the deep nuclei, under the external capsule and claustrum, it travels in contiguity with optic radiations and reaches the occipital cortex (Figure 5D).

### The Ovine Brain Tractography Atlas

**FIGURE 6:**
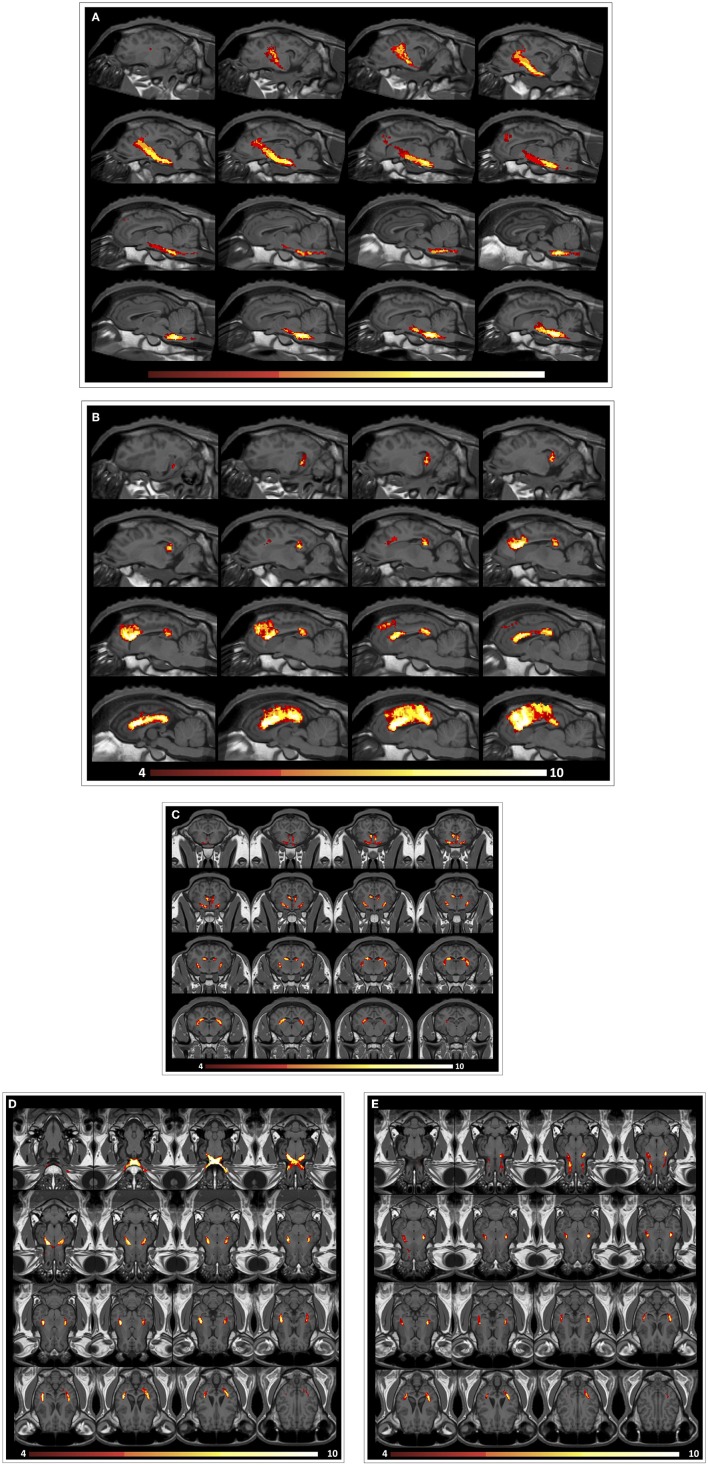
Representative images from the ovine tractographic atlas. The tractography atlas of the five ovine tracts is visualized by means of FSLeyes in sagittal view for CST **(A)** and CC **(B)**, transverse view for FX **(C)**, and coronal view for VP **(D)** and OF **(E)**, overlaid onto the publicly available stereotaxic T1-weighted ovine brain atlas 0.5 mm. The origin of the reference system with the xyz-values (0;0;0) is a vertical line perpendicularly intersecting the superior aspect of the rostral commissure. All coordinates are given in millimeters (mm). Values of the x-axis increase from left to right, values of the y-axis increase from rostral to caudal, while values of the z-axis increase in the dorsal direction (31). **(A)** sagittal view for CST: on x axis, from −12.9 to 5.1 mm (slice spacing: 1.2 mm). **(B)** sagittal view for CC: on x axis, from −14.6 to 3.4 mm (slice spacing: 1.2 mm). **(C)** transverse view for FX: on y axis, from 3.65 to −17.35 mm (slice spacing: 1.4 mm). **(D)** coronal view for VP: on z axis, from −11.1 to 15.9 mm (slice spacing: 1.8 mm). **(E)** coronal view for OF: on z axis, from −6.225 to 17.025 mm (slice spacing: 1.55 mm). By fixing the cutoff value at 4, only the fibers that are consistent in at least 4 sheep out of 10 are shown.

**FIGURE 7:**
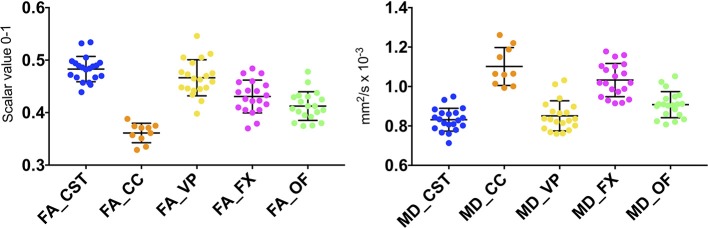
Scatterplots displaying FA and MD in the five reconstructed fiber bundles. Color-coding of the scatterplots corresponds to Figures 4, 5. Blue=CST; orange=CC; yellow=VP; fuchsia=FX; green=OF). Black bars show the mean and SD between 10 CCs and 20 CSTs, VPs, FXs, OFs (10 right and 10 left), each one represented by a single dot.

An ovine brain tractography atlas was successfully obtained by combining every single WM tract of the 10 animals. Figure 6 shows the resulting probability maps for each tract superimposed to the publicly available T1-weighted stereotaxic ovine brain template (31). Representative slices are displayed, showing the main course of each tract in all the 10 animals (Figures 6A–E). Tract masks are color-encoded according to the number of animals in which the tract passes through each voxel. Voxel values range from 1 (meaning that the voxel is occupied by the tract of a single sheep) to progressive numbers the more the tract is consistent between the animals, up to a value of 10 in voxels occupied by the tracts of all the 10 sheep. The complete set of probability maps throughout the whole the rostro-caudal extent of the atlas is available for download at https://www.eden2020.eu/data-sets/. Intensity values can be visualized in gray or in color scales with FSLeyes (https://fsl.fmrib.ox.ac.uk/fsl/fslwiki/FSLeyes). As an example, in Figure 6 the intensity values are shown in color scales, excluding the voxels less consistent between the animal cohort. In fact, fixing the cutoff at 4 allows to visualize only the fibers that are consistent in at least 4 sheep out of 10. Eventually, the population-averaged FA and MD values were estimated for each reconstructed tract. Diffusion metrics of white matter fibers of the 10 sheep are summarized in Table 1 and displayed in boxplots in Figure 7. Minimum and maximum values quantified in each tract among our sample were reported, as well as standard deviations, referring to the inter-subject variability. As it could be expected, the MD and FA values are quite variable between different tracts, while the variability among sheep is minimal.

**Table 1:** Descriptive statistics of quantitative diffusion metrics in fiber tracts.

**Tract**	**Fractional anisotropy (FA: scalar value 0-1)**			**Mean diffusivity (MD: mm**^****2****^**/s** **×** **10**^****−3****^**)**		
	**Minimum**	**Maximum**	**Mean ± SD**	**Minimum**	**Maximum**	**Mean ± SD**
Corticospinal tract (CST)	0.439	0.534	0.483 ± 0.024	0.713	0.949	0.832 ± 0.058
Corpus callosum (CC)	0.329	0.388	0.361 ± 0.019	0.993	1.261	1.102 ± 0.096
Visual pathway (VP)	0.398	0.576	0.489 ± 0.046	0.760	1.032	0.880 ± 0.081
Fornix (FX)	0.370	0.484	0.431 ± 0.031	0.915	1.178	1.033 ± 0.084
Occipitofrontal fasciculus (OF)	0.374	0.478	0.412 ± 0.027	0.807	1.052	0.908 ± 0.066

*Each row corresponds to a tract; columns display the minimum value, maximum value and mean± SD of FA and MD measured among the 10 sheep*.

## Discussion

In this work we demonstrate the feasibility of a protocol to perform *in vivo* DTI tractography of the sheep model, providing a reliable reconstruction and 3D rendering of major ovine fiber tracts responsible for different functions. The main novelty of the present study is the creation of a comprehensive population-averaged sheep tractography atlas, that aims at assessing ovine white matter anatomical distribution. The probability map method allowed us to efficiently overlap fiber bundles belonging to 10 different sheep, with the final purpose of appreciating inter-subject variability and highlighting the tract core, common to all the animals. A detailed pipeline for DTI data acquisition, preprocessing and analysis, as well as for the identification of seed ROIs for tractography, has been implemented in order to reconstruct the location and trajectory of five eloquent WM tracts representative of the main fiber bundles included in typical *in vivo* DTI studies, namely the corticospinal tract (CST), corpus callosum (CC), visual pathway (VP), fornix (FX), and occipitofrontal fasciculus (OF). In fact, exclusively the ovine motor pathway (33, 44) and optic radiations (33) have been previously described in DTI studies on the sheep model, with a limited number of subjects.

Our analysis improved the anatomical understanding of the normal appearance of these ovine WM bundles and allowed a comparison to the homologous structures of human and other mammals, which is pivotal for the translational purpose of using sheep models in neuroscience. Indeed, WM fibers have been conventionally classified into different categories depending on their paths, both in humans and in animals (17, 18). For instance, tracts in the brainstem comprise the motor fibers of the CST and its cerebellar connections, while projection fibers include the suprathalamic portion of CST that connects cortical to subcortical white matter. With respect to the other primates, the CST in sheep is smaller and composed of thinner fibers (40). It is important to highlight that, besides actual CST fibers, the projections from the internal capsule to the pons revealed by our DTI analyses may include other longitudinal tracts passing through the brainstem. Given that DTI virtual dissections have lower resolution than real anatomical dissections and that our ROIs have been manually contoured, it cannot be excluded that part of corticofugal fibers, corticonuclear fibers, or even extrapyramidal tracts has ended up depicted in our bundles labeled as CSTs. In order to avoid false positives, the most consistent CST portion can be appreciated in the atlas, thresholding images to visualize just those voxels where CST resulted common to all the animals. Another interesting aspect is that the motor cortex is mesial in the ovine, constituted by the precruciate gyrus that is located before the cruciate fissure. The latter corresponds to the human fissure of Rolando or central sulcus, and intersects the medial longitudinal fissure to mark off the anterior third of the cortex (43). Due to the para-sagittal disposition of the motor cortex, along the longitudinal fissure, the sheet of the corona radiata can be appreciated on the sagittal plane. Conversely, the human motor cortex extends along the central sulcus in the precentral gyrus, thus the corona radiata fans out in an arc on the coronal plane (41). A further white matter category includes commissural tracts that connect the right and left hemispheres and are responsible both for homologous and heterotopic associations, as the CC. It consists of a flat bundle of fibers both in sheep and humans, spanning part of the longitudinal fissure (38). Additional eloquent tracts that we managed to reconstruct in the ovine model are the VP for the eyesight and the FX, that is part of the limbic system. In the ovine VP, the same components that carry visual information from the environment to the brain in humans are identifiable, including the optic nerve, optic chiasm, optic tract, lateral geniculate nucleus, optic radiations, and visual cortex, located in the occipital lobe as already described in the literature (36). Notably, the percentage of fibers that do not cross in the contralateral optic tract represents a recent filogenetic acquisition that allows a stereoscopic vision, thus it reaches only 11% in ovine, while 47% in humans (38). Moreover, in sheep, a small supplementary bundle of fibers defined as *fasciculus paraopticus* runs along the medial margin of the optic tract. It groups fibers coming from the contralateral retina and can be considered an isolated portion of the medial root of the optic tract (38). As far as the FX is concerned, it appears particularly trophic in sheep and emerges from the hippocampus as a C-shaped fiber bundle. It carries fibers from the hippocampus to the mammillary bodies (via the post-commissural fornix) and septal nuclei (via the precommissural fornix), and fibers from septal nuclei to hippocampus, thus seeming associated to sheep memory formation (38), exactly as in humans. Eventually, a further category of WM bundles is represented by the associative fibers, that connect two different cortical areas. In humans, they include the Inferior Fronto Occipital Fascicle, Uncinate Fascicle, Cingulum, Inferior Longitudinal fascicle, Superior Longitudinal Fascicle and Arcuate Fascicle, and are responsible for higher functions such as language production and comprehension. Since associative fibers have been described in dogs (17, 18) bovines (45), and dolphins (46), we expected to find the OF also in sheep, even if its precise function has not been completely elucidated in animals.

Our study also aimed at evaluating the reproducibility of the sheep tractography pipeline. To this end, it was firstly figured out that the ROIs identified as appropriate seeds for the tracking algorithm could be consistently contoured in every animal, by taking into account an accurate examination of the prior knowledge on sheep neuroanatomy (36–40), human DTI atlas (41, 42), and gross dissections of ovine brain (43). At a qualitative, visual assessment the course of tracts appears very similar across the 10 animals, thus highlighting the reproducibility of the tracking pipeline, adding strength and consistency to our findings.

Furthermore, in order to precisely quantify inter-sheep similarity and to provide an information regarding the average position of each tract and the normal variability across sheep, the 10 individual MRI datasets were separately analyzed and then coregistered to a publicly available stereotaxic T1-weighted ovine brain atlas (31) in a standard coordinate system. A population-averaged atlas representing the five most eloquent white matter ovine fiber bundles was generated, providing a standard sheep brain tractography template that can be easily overlaid onto the aforementioned reference space. As confirmed by many researchers (30), the possibility to integrate our tractography reconstructions into other publicly available databases is of striking significance, since the availability of a common reference space for standardization is the basis for any future significant neuroimaging study. In fact, the atlas would offer to veterinaries and researchers the possibility to incorporate tractography in the study of numerous brain diseases in the ovine translational models, even if DTI acquisitions are not available. For example, Staudacher et al. pointed out that new technologies and tools are needed to improve the accuracy in brainstem biopsies essential for histopathological diagnoses (47), and the atlas could be fundamental to this purpose, precisely locating the CST pyramids. Furthermore, despite the possibility to identify ischemic injuries in ovine white matter by means of conventional MRI (48, 49), tractography would add specificity to the study of the complex pathophysiology of white matter damages, allowing researchers to focus their analyses on the core of the fiber bundles. From a more general perspective, then, the tractography atlas will facilitate the localization of different sheep cortical areas, implementing future studies on acute mapping procedures and possibly allowing the recording of motor potentials besides the sensory ones elicited by Gierthmuehlend et al. in a recent study (50).

Key features of the proposed tractography atlas include also its versatility and adaptability to various clinical contexts. Indeed, the atlas consists in masks of white matter tracts that are not binary, but that are weighted from 1 to 10 according to the number of fiber bundles present in every specific voxel. The comprehensive tract masks that we generated for CST, CC, VP, FX, and OF, in fact, derived from the sum of the corresponding fibers of 10 individual sheep previously coregistered to the same reference space. Therefore, their central core resulted common to all the animals, while subtle anatomical variations could be appreciated for more external fibers. Specifically, all the comprehensive masks can be thresholded for any value from 1 to 10 according to particular experimental needs, so to visualize and consider different levels of inter-subject variability. As an example, in the field of neurosurgical research, presurgical planning on sheep must be precisely tuned depending on the final aim of the procedure, both in the clinical veterinary routine as well as in experimental settings, in order to faithfully reproduce the human patient scenario. On one hand, if the ultimate goal of the surgery is to precisely target WM tracts with electrical stimulation, the operator would probably want to consider only fibers that we tracked in 8–10 out of the total of 10 sheep, to rely just on the tract portion more preserved across different animals. The study conducted by Stypulkowski et al. represents a fitting example in which the tractography atlas would have been valuable, since they stimulated the Papez circuit with deep brain stimulation (DBS) targeting the fornix only on the basis of anatomical notions, without the specificity of tractography reconstructions (51).

On the other hand, if the final purpose of the procedure is to safely remove a mass lesion without compromising the sheep's quality of life, the operator would probably prefer to consider all the possible fibers passing through the area of interest, to remain more conservative and spare eloquent structures. For example, the atlas could intriguingly implement the cerebral tumor model based on the injection of agar into sheep brain, proposed by Kamp et al. for the training of young neurosurgeons (52). In this case, the mask threshold can be fixed around 3, in order to visualize white matter fibers present in at least 3 sheep out of the total of 10, to compute a realistic integration of WM obstacles in the simulation setting and to challenge neurosurgeons in sparing pivotal functions such as movement and vision. Ultimately, regardless of the context-dependent mask-threshold chosen by the operator, a detailed representation of white matter structures is essential for the study of many brain pathologies, from developmental ones to degenerative ones, so the exploitation of our atlas could be precious in several contexts, both for translational research in humans and in the veterinary application *per se*.

Focusing on the quantitative estimates of diffusion metrics, the population-averaged values of MD and FA provided in Table 1 for the five considered tracts can be used as reference values for the healthy ovine brain in preclinical studies. While the tractographic atlas describes the structure and spatial distribution of the fiber bundles, the diffusion metrics are informative of microstructural tissue characteristics. The significance of the two information is therefore complementary, and alterations in either or both these features may reveal underlying pathological processes.

A potential limitation of this study can be represented by the restricted number of animals included. However, considering that *in vivo* MRI studies on sheep are multifaceted and demanding, as highlighted also by other studies (10, 33), a sample size of 10 animals can already be considered valuable. Another possible concern relies on the clinically-compatible MRI acquisitions on a 1.5T scanner, that may impede to depict fine anatomical details of the tracts. Recent studies have exploited DTI at 3T for tractography of white matter tracts of large animal models (17, 33, 46). However, at present, 3T MR scanners totally dedicated to veterinary imaging are rarely available in Italy and Europe, mainly due to the equipment disproportionate costs with respect to the clinical routine of veterinary practice. Nonetheless, in our study high-quality images have been obtained in clinically compatible scanning duration, without the need of keeping the sheep anesthetized for long time. Both diffusion maps and tractography reconstructions have been feasible with our datasets, and can be easily implemented in preclinical studies aimed at evaluating multiple time-points *in vivo* in an experimental setting resembling a clinical scenario.

Finally, the possible inaccuracy derived from anatomical variability of the animals may be improved even considering other ovine breeds. Future investigation will be aimed at exploring the degree of heterogeneity in brain anatomy between different ovine varieties, possibly leading toward a more accurate representation of pivotal neuroanatomical structures in exceptionally detailed atlases (53).

## Conclusion

The present work built an innovative ovine tractography atlas, demonstrating that multiple white matter fiber tracts can be consistently reconstructed in sheep. Minimal inter-subject variability proves the reproducibility of our image post-processing, ROIs identification and fiber tracking. Additionally, the population-averaged atlas can be integrated into publicly available imaging software, paving the way toward space standardization of ovine imaging analyses. It will enable to design homogeneous studies considering the direction and reciprocal position of white matter fiber bundles, that will significantly support the meticulous study of numerous brain pathologies. In conclusion, the ovine tractography atlas can be considered as a valuable tool to implement the knowledge of sheep's brain anatomy and to improve the activity of clinicians and researchers using this animal model in neuroscience studies.

## Data Availability Statement

The datasets generated for this study are available on request to the corresponding author.

## Ethics Statement

The animal study was reviewed and approved by Ethical approval for this study was obtained by the Italian Health Department with authorization n 635/2017.

## Author Contributions

AC, AF, MR, and LB contributed conception and design of the study. MT, SB, DZ, GR, FA, and MG guaranteed the animal well-being, transport and anesthesia. MC, AC, LM, and MD optimized the MRI protocol. AC, VP, and LM acquired the MRI dataset.VP and MT performed reconstructions and analyses. VP and AC wrote the first draft of the manuscript. MT wrote sections of the manuscript. All authors contributed to manuscript revision, read, and approved the submitted version.

### Conflict of Interest

MC was employed by company Philips Healthcare, Italy. The remaining authors declare that the research was conducted in the absence of any commercial or financial relationships that could be construed as a potential conflict of interest.
