# Supplementary material for: In vivo Diffusion Tensor Magnetic Resonance Tractography of the Sheep Brain: An Atlas of the Ovine White Matter Fiber Bundles
Source: Front Vet Sci. 2019 Oct 16;6:345. doi: 10.3389/fvets.2019.00345 (PMC6805705; doi:10.3389/fvets.2019.00345)
Supplement: Supplementary file 1 [file Data_Sheet_1.docx]

Supplementary Material

# Supplementary Tables

## Supplementary Table 1 – Example of the detailed ethogram

For all sheep, a dedicated ethogram was daily filled in during the entire housing period. Detailed scores for clinical and physiological parameters were reported in order to guarantee the animal wellbeing and to intervene if the animal balance is compromised.

| **Date**  **Sheep n°**  **Weight** | **Score** | **Date/Hour** |
| --- | --- | --- |
| **Sensorium** | 0/3 |  |
| **Eating** | Y/N |  |
| **Ruminating** | Y/N |  |
| **Drinking** | Y/N |  |
| **Lameness** | 0-3 |  |
| **Rectal Temperature**  **(39-40°C)** | 0/3 |  |
| **Respiratory frequency**  **(12-20 breaths per min)** | 0/3 |  |
| **Cardiac frequency**  **(70-80 beat per min)** | 0/3 |  |
| **Body condition score (BCS)*** | 0/5 |  |

Table legend on animal welfare (degree of severity):

- Sensorium: Normal: 0 = no grade (normal behaviour), 1= low activity (reduction of motility, lethargy), 2 = no activity (stillness or reluctance to move), 3 = persistent decubitus or coma
- Lameness: 0 = no grade, 1 = mild, 2 = moderate, 3= severe
- Body temperature: 0 = normothermia, 1 = hypothermia, 2 = hyperthermia
- Respiratory frequency: 0 = eupneic, 1 = bradypneic, 2 = tachypneic, 3 = dyspneic
- Cardiac frequency: 0 = normocardia 1= bradycardia, 2=tachycardia, 3=tachyarrhythmia
- Y/N= YES/NO
- *BSC: 0 = emaciated, 1 = very thin, 2 = thin, 3 = moderate (ideal), 4 = fleshy, 5 = very fleshy

## Supplementary Table 2 – Complete MRI protocol acquired in sheep

Technical parameters of the MR sequences acquired in sheep are herein reported.

|  | **3D T1** | **T2** | **SWI** | **PCA** | **TOF HR** | **DTI15** |
| --- | --- | --- | --- | --- | --- | --- |
| **Sequence** | sT1w_3D_FFE | T2W_TSE | SWIp | s3D_PCA_SAG | TOF | DTI15 |
| **TR (ms)** | 25 | 7557 | 52 | 12 | 25 | 6700 |
| **TE (ms)** | 5 | 110 | 0 | 7 | 7 | 84 |
| **Flip Angle** | 40 | 90 | 20 | 10 | 20 | 90 |
| **Acquisition Matrix** | 288x288 | 512x512 | 192x192 | 256x256 | 320X320 | 96x96 |
| **Voxel size (mm)** | 0.667x0.667 | 0.314x0.314 | 1x1 | 0.898x0.898 | 0.562x0.562 | 2x2 |
| **Slice Thickness**  **(mm)** | 1.4 | 3 | 2 | 1.6 | 1.2 | 2 |
| **Slice number** | 150 | 34 | 56 | 76 | 120 | 45 |
| **SENSE factor** | 2 | 1 | 2 | 1.8 | 2 | 2 |
| ***b*-value**  **(s/mm^2^)** | - | - | - | - | - | 0  1000 (15 dir) |
| **Acquisition Time** | 8 min 40 sec | 5 min 41 sec | 4 min 34 sec | 3 min 52 sec | 4 min 5 sec | 5 min 34 sec |

**
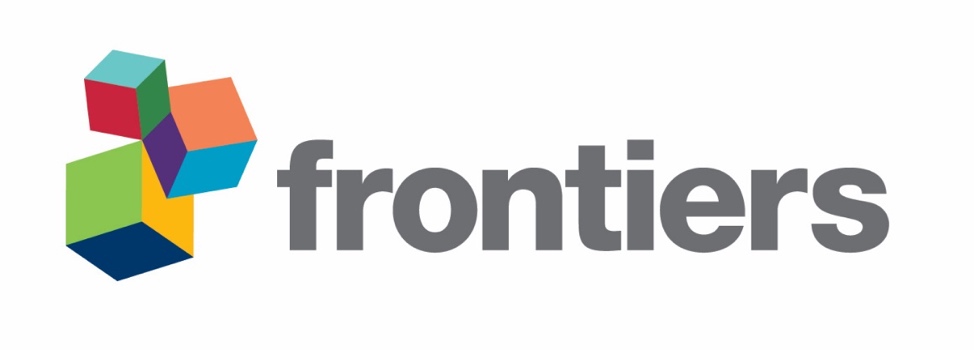
**
